# Physical comorbidities increase the risk of psychiatric comorbidity in multiple sclerosis

**DOI:** 10.1002/brb3.493

**Published:** 2016-06-29

**Authors:** Ruth Ann Marrie, Scott B. Patten, Jamie Greenfield, Lawrence W. Svenson, Nathalie Jette, Helen Tremlett, Christina Wolfson, Sharon Warren, Joanne Profetto‐McGrath, John D. Fisk, the CIHR Team in the Epidemiology and Impact of Comorbidity on Multiple Sclerosis, James Blanchard, Patricia Caetano, Lawrence Elliott, Bo Nancy Yu, Virender Bhan, Larry Svenson

**Affiliations:** ^1^ Department of Internal MedicineUniversity of ManitobaWinnipegManitobaCanada; ^2^ Department of Community Health SciencesUniversity of ManitobaWinnipegManitobaCanada; ^3^ Department of Community Health SciencesUniversity of CalgaryCalgaryAlbertaCanada; ^4^ Department of Clinical Neurosciences and Hotchkiss Brain InstituteUniversity of CalgaryCalgaryAlbertaCanada; ^5^ School of Public HealthUniversity of AlbertaEdmontonAlbertaCanada; ^6^ Surveillance and AssessmentAlberta HealthEdmontonAlbertaCanada; ^7^ O'Brien Institute for Public HealthUniversity of CalgaryCalgaryAlbertaCanada; ^8^ Department of Medicine (Neurology)University of British ColumbiaVancouverBritish ColumbiaCanada; ^9^ Department of Epidemiology and Biostatistics and Occupational HealthMcGill UniversityMontrealQuebecCanada; ^10^ Faculty of Rehabilitation MedicineUniversity of AlbertaEdmontonAlbertaCanada; ^11^ Faculty of NursingUniversity of AlbertaEdmontonAlbertaCanada; ^12^ Departments of Psychiatry and MedicineDalhousie UniversityHalifaxNova ScotiaCanada

**Keywords:** administrative data, anxiety, comorbidity, depression, multiple sclerosis

## Abstract

**Background:**

Risk factors for psychiatric comorbidity in multiple sclerosis (MS) are poorly understood.

**Objective:**

We evaluated the association between physical comorbidity and incident depression, anxiety disorder, and bipolar disorder in a MS population relative to a matched general population cohort.

**Methods:**

Using population‐based administrative data from Alberta, Canada we identified 9624 persons with MS, and 41,194 matches. Using validated case definitions, we estimated the incidence of depression, anxiety disorder, and bipolar disorder, and their association with physical comorbidities using Cox regression, adjusting for age, sex, socioeconomic status, and index year.

**Results:**

In both populations, men had a lower risk of depression and anxiety disorders than women, as did individuals who were ≥45 years versus <45 years at the index date. The risk of bipolar disorder declined with increasing age. The risks of incident depression (HR 1.92; 1.82–2.04), anxiety disorders (HR 1.52; 1.42–1.63), and bipolar disorder (HR 2.67; 2.29–3.11) were higher in the MS population than the matched population. These associations persisted essentially unchanged after adjustment for covariates including physical comorbidities. Multiple physical comorbidities were associated with psychiatric disorders in both populations.

**Conclusion:**

Persons with MS are at increased risk of psychiatric comorbidity generally, and some physical comorbidities are associated with additional risk.

## Introduction

Psychiatric comorbidity, including depression, anxiety disorders, and bipolar disorder affects the multiple sclerosis (MS) population more often than the general population (Marrie et al. 2015a). Psychiatric comorbidity is associated with increased hospitalizations, increased mortality, and lower health‐related quality of life in MS (Marrie et al. 2015b,c). Psychiatric comorbidity is also associated with poor adherence to therapy (Mohr et al. 1997). Therefore, a better understanding of risk factors for psychiatric comorbidity in MS is needed.

Chronic medical conditions are often associated with an increased burden of psychiatric comorbidity (Wells et al. 1988; Egede 2007; Eaton et al. 2010; Hsu et al. 2014). The presence of other medical conditions (i.e., physical comorbidity) is common in MS (Marrie et al. 2015a) and one study suggested that multiple physical comorbidities independently increased the risk of depression in MS (Marrie et al. 2009). However, that study was not population‐based, and did not establish whether the association of physical comorbidity and depression differed from expectations for the general population (Marrie et al. 2009). It also did not evaluate the association of physical comorbidity with psychiatric comorbidities other than depression, even though other psychiatric conditions often co‐occur with depression and contribute to poor outcomes (Marrie et al. 2015b,c).

We used population‐based administrative data to evaluate the association of physical comorbidity with incident psychiatric comorbidity, including depression, anxiety disorders, and bipolar disorder in the MS population and in a matched cohort from the general population. We hypothesized that the increased risk of psychiatric comorbidity in the MS population as compared to the matched population would be at least partially accounted for by increased physical comorbidities in the MS population.

## Materials and Methods

### Data

We used administrative data from the province of Alberta, Canada for the period 1 April 1994–31 March 2011. Alberta Health administers health services to the entire provincial population, and maintains records related to health service delivery including a population registry, hospital discharge abstracts, and physician (medical services) visits. The population registry includes sex, and dates of birth, death, and health care coverage. Hospital discharge abstracts include dates of admission and discharge, and up to 25 discharge diagnoses coded using the International Classification of Diseases (ICD) system (ICD‐9‐CM until March 31, 2002; ICD‐10‐CA coding thereafter). Physician visits include the service date, and up to three diagnoses using ICD‐9‐CM codes. We obtained ethics approval from the University of Calgary, and approval to use the administrative data from Alberta Health.

### Study populations

To identify all persons with MS in Alberta, we applied a validated administrative case definition (Marrie et al. 2012), requiring ≥3 (hospitalizations or physician visits) for MS as identified using an ICD‐9/10 code of 340/G35 (*n* = 13,531) during the period 1994–2011. We excluded individuals <20 years old (*n* = 212), or with any gap in follow‐up between first identification in the population registry and the index date (*n* = 417). Subsequently, we identified a general population cohort. First, we excluded anyone with ICD‐9/10 codes for demyelinating disease (377.3/H46, 323.82/G37, 323/G36.9, 341.9/G37.8, G36, 340/G35, or 341.0/G36.0). Second, we identified controls matched on sex, exact year of birth, and region of residence (postal code). We randomly selected up to five controls per case (*n* = 67,505). For each person with MS, we assigned the date of the first health claim for demyelinating disease as the “date of diagnosis”, and assigned the same (index) date to the matched controls. We could not match 30 individuals with MS.

### Identifying physical and psychiatric conditions

The considered physical conditions included those associated with psychiatric disorders in either MS (diabetes, hypertension, hyperlipidemia, fibromyalgia, chronic lung disease, inflammatory bowel disease [IBD]) (Marrie et al. 2009), rheumatoid arthritis, another immune‐mediated inflammatory disease (chronic lung disease) (Hsu et al. 2014), or diabetes, another common chronic condition (ischemic heart disease [IHD]) (Egede 2005). We limited our selection of physical conditions to those with a validated algorithm that could identify them from administrative data (Marrie et al. 2012, 2013a,b,c). Validated algorithms (Marrie et al. 2012, 2013a,b,c) were applied to identify depression, anxiety disorders, and bipolar disorder, as well as chronic lung disease, diabetes, epilepsy, fibromyalgia, hyperlipidemia, hypertension, IBD, and IHD. Because all physical comorbidities were chronic conditions, once an individual met the case definition for a condition, they were considered affected thereafter if alive and residing in Alberta.

To identify incident cases of depression, anxiety disorders, and bipolar disorder, we required a 5‐year run‐in period before the first relevant claim. Therefore, incidence rates were estimated for 1999–2011. Comorbidities were considered incident if the first claim occurred following the date of MS diagnosis (or corresponding index date for the matched cohort). We excluded individuals with any gap in follow‐up between first identification in the population registry and the index date (dropped 3665 cases and 25,223 controls).

### Analysis

First, we estimated the incidence of depression, anxiety disorder, and bipolar disorder in both populations, calculating 95% confidence intervals (CI) assuming a Poisson distribution. Consistent with earlier work (Marrie et al. 2015d), we age‐standardized the findings to the 2001 Canadian population. Second, we evaluated the association between physical comorbidity and the risk of developing psychiatric comorbidity using multivariable Cox proportional hazards models. Zero time was the MS diagnosis date, and the study endpoint was the incident psychiatric comorbidity, death, emigration from Alberta, or the study end, whichever came first. Physical comorbidities were included as time‐varying variables; the first claim for the comorbidity of interest defined the onset of exposure, and all subsequent person‐time was classified as exposed. Covariates were sex (female as reference group), age at the index date (20–44 [reference group], 45–59, ≥60 years), year of diagnosis (1994 [reference group], 1995–1998, 1999–2002, 2003–2006, ≥2007), and socioeconomic status (SES). SES was defined in quintiles through linkage to the 2006 Canadian census, which provided median household income by forward sortation area (first three digits of postal code); the lowest quintile was the reference group. Analyses were conducted separately for depression, anxiety disorders, and bipolar disorder. We tested for the presence of interactions between study population and (i) sex, (ii) physical comorbidities; and between sex and physical comorbidities. We present hazard ratios (HR) and 95% confidence intervals.

Statistical analyses used SAS V9.3 (SAS Institute Inc., Cary, NC). For each psychiatric disorder, we controlled the false discovery rate for comorbidity using the Benjamini–Hochberg procedure.

## Results

### Study populations

**Figure 1:**
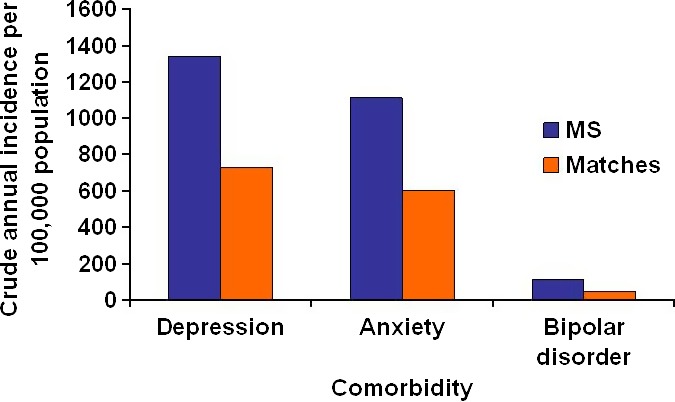
Annual incidence per 100,000 persons of psychiatric comorbidity in the MS and matched populations in 1999.

We identified 9624 MS cases and 41,194 matched controls (Table 1). In 1999, the crude annual incidence of depression, anxiety disorders, and bipolar disorder were higher in the MS population than in the matched population (Fig. 1). These associations were consistent for 1999–2011 (Fig. S1‐S3). Tables S1 and S2 show age‐specific and sex‐specific average annual incidence rates of depression, anxiety disorders, and bipolar disorder. The incidence of depression and anxiety disorders were higher among women than men in both populations, but the incidence of bipolar disorder did not differ by sex.

**Table 1:** Characteristics of multiple sclerosis population and matched cohort from general population

Characteristic	Cases (*N* = 9624)	Controls (*N* = 41,194)	*P*‐value
Duration of follow‐up from index date to study end date (years), mean (SD)	10.3 (5.7)	9.98 (5.5)	<0.0001
Duration of follow‐up from index date to endpoint date (years), mean (SD)	9.76 (5.7)	8.95 (5.6)	0.0005
Sex, *n* (%)			
Female	6635 (68.9)	27,595 (67.0)	<0.0001
Male	2989 (31.1)	13,599 (33.0)	
Age (years), mean (SD)	42.9 (13.3)	42.8 (13.3)	0.51
Age Group (years), *n* (%)			
20–44	5701 (59.2)	24,458 (59.4)	0.93
45–59	2799 (29.1)	11,982 (29.1)	
≥60	1124 (11.7)	4754 (11.5)	
Income Quintile, *n* (%)^a^			
Quintile 1	1911 (20.0)	8110 (19.8)	0.97
Quintile 2	2585 (27.0)	11,048 (27.0)	
Quintile 3	1816 (19.0)	7710 (18.8)	
Quintile 4	1737 (18.1)	7549 (18.4)	
Quintile 5	1526 (15.9)	6555 (16.0)	
Physical Comorbidity, *n* (%)			
Diabetes	222 (2.3)	1160 (2.8)	0.006
Hypertension	684 (7.1)	3003 (7.3)	0.55
Ischemic Heart Disease	157 (1.6)	830 (2.0)	0.02
Hyperlipidemia	364 (3.8)	1871 (4.5)	0.001
Chronic Lung Disease	572 (5.9)	2256 (5.5)	0.076
Fibromyalgia	118 (1.2)	172 (0.42)	<0.0001
Epilepsy	115 (1.2)	177 (0.43)	<0.0001
Inflammatory Bowel Disease	30 (0.31)	75 (0.18)	0.02
Index Year, *n* (%)			
1994	2423 (25.2)	10,497 (25.5)	0.81
1995–1998	2334 (24.3)	10,089 (24.5)	
1999–2002	1851 (19.2)	7958 (19.3)	
2003–2006	1562 (16.2)	6515 (15.8)	
2007–2011	1454 (15.1)	6135 (14.9)	

SD, Standard deviation.

a Missing in 271 (49 MS cases + 222 matched controls).

### Physical comorbidity and depression

On univariate analysis, the risk of incident depression was higher in the MS population than in the matched population (HR 1.97; 1.86–2.08). This association persisted after adjusting for age, sex, index year, SES, and each physical comorbidity (HR 1.92; 1.82–2.04). In both populations, men had a lower risk of depression than women, as did individuals who were ≥45 years versus <45 years at the index date (Table 2).

**Table 2:** Adjusted hazard ratios and 95% confidence intervals (HR) for the association between physical comorbidity^a^ and depression in the multiple sclerosis (MS) and matched populations

Variable	Both populations^b^ (*N* = 49,069)	Matched population only^c^ (*N* = 39,698)	MS population only^c^ (*N* = 9105)
Population			
Matched	1.0	1.0	1.0
MS	**1.92 (1.82**–**2.04)**	–	–
Sex			
Female	1.0	1.0	1.0
Male	**0.66 (0.62**–**0.70)**	**0.62 (0.58**–**0.66)**	**0.78 (0.70**–**0.87)**
Age at index date (years)			
20–44	1.0	1.0	1.0
45–59	**0.73 (0.69**–**0.78)**	**0.73 (0.67**–**0.78)**	**0.76 (0.67**–**0.85)**
≥60	**0.84 (0.77**–**0.92)**	**0.88 (0.79**–**0.97)**	**0.78 (0.70**–**0.87)**
Index year			
1994	1.0	1.0	1.0
1995–1998	**1.21 (1.13**–**1.29)**	**1.16 (1.08**–**1.25)**	**1.37 (1.21**–**1.56)**
1999–2002	**1.50 (1.40**–**1.62)**	**1.39 (1.28**–**1.52)**	**1.84 (1.60**–**2.12)**
2003–2006	**1.44 (1.31**–**1.59)**	**1.35 (1.21**–**1.52)**	**1.70 (1.43**–**2.03)**
≥2007	**1.92 (1.67**–**2.20)**	**1.65 (1.38**–**1.97)**	**2.59 (2.07**–**3.24)**
Socioeconomic status			
Quintile 1	1.0	1.0	1.0
Quintile 2	1.01 (0.94–1.09)	0.96 (0.88–1.04)	1.14 (0.99–1.31)
Quintile 3	1.02 (0.94–1.11)	1.04 (0.95–1.14)	0.97 (0.83–1.13)
Quintile 4	1.07 (0.99–1.16)	1.02 (0.93–1.13)	1.19 (1.03–1.39)
Quintile 5	0.99 (0.91–1.08)	0.97 (0.87–1.07)	1.05 (0.89–1.24)
Physical comorbidity			
Diabetes	**1.16 (1.05**–**1.28)**	**1.15 (1.03**–**1.29)**	1.18 (0.96–1.45)
Hypertension	**1.16 (1.08**–**1.25)**	**1.15 (1.06**–**1.25)**	**1.16 (1.01**–**1.33)**
Ischemic heart disease	**1.56 (1.40**–**1.72)**	**1.61 (1.43**–**1.81)**	**1.38 (1.11**–**1.73)**
Hyperlipidemia	**1.10 (1.02**–**1.19)**	**1.12 (1.03**–**1.23)**	0.99 (0.84–1.18)
Lung disease	**1.51 (1.41**–**1.63)**	**1.51 (1.39**–**1.64)**	**1.50 (1.31**–**1.72)**
Fibromyalgia	**1.87 (1.61**–**2.17)**	**2.05 (1.69**–**2.48)**	**1.67 (1.32**–**2.12)**
Epilepsy	**1.75 (1.48**–**2.06)**	**1.97 (1.55**–**2.50)**	**1.64 (1.31**–**2.05)**
Inflammatory bowel disease	**1.63 (1.19**–**2.21)**	**1.62 (1.11**–**2.36)**	1.54 (0.91–2.61)

a All physical comorbidities included simultaneously in the multivariable models.

b Main model. All main effects for comorbidity statistically significant after using Benjamini‐Hochberg correction. Interactions between population and ischemic heart disease, hyperlipidemia also statistically significant.

c Stratified models illustrate interaction effects.

Bold text indicates findings that are statistically significant.

Due to an interaction between sex and population (*χ* ^2^ = 11.4, *P* = 0.0007), we stratified the analysis. Men in the MS population had a 22% reduced hazard of incident depression versus women; however, men in the matched general population had a 38% reduced hazard of depression (Table 2). We also observed interactions between population and IHD (*χ* ^2^ = 6.32, *P* = 0.01), and hyperlipidemia (*χ* ^2^ = 4.37, *P* = 0.04). IHD was more weakly associated with depression in the MS population than in the matched population while hyperlipidemia was not associated with depression in the MS population. However, hypertension, lung disease, fibromyalgia, and epilepsy were associated with depression in both populations. When the model combined both populations, diabetes and IBD were also associated with depression. Although we did not detect an interaction between population and these latter conditions, the association of diabetes and IBD with depression did not reach statistical significance in the MS population alone, possibly due to smaller sample sizes.

We observed interactions between sex and diabetes (*χ* ^2^ = 5.72, *P* = 0.02), hypertension (*χ* ^2^ = 4.94, *P* = 0.03), and chronic lung disease (*χ* ^2^ = 4.55, *P* = 0.03); with the risk of depression conferred by these comorbidities greater in men than women (Table S3). In the stratified analysis these findings persisted for diabetes and hypertension in the matched population only.

### Physical comorbidity and anxiety disorders

On univariate analysis, the risk of incident anxiety disorders was higher in the MS population than in the matched population (HR 1.55; 1.45–1.66). This association persisted after accounting for covariates including physical comorbidities (HR 1.52; 1.42–1.63). As observed for depression, men had a lower risk of anxiety disorders than women, as did individuals who were ≥45 years versus <45 years at the index date (Table 3).

**Table 3:** Adjusted hazard ratios and 95% confidence intervals (HR) for the association between physical comorbidity^a^ and anxiety disorder in the multiple sclerosis (MS) and matched populations

Variable	Both populations (*N* = 48,973)^b^	Matched population only^c^ (*N* = 39,603)	MS population only^c^ (*N* = 9105)
Population			
Matched	1.0	–	–
MS	**1.52 (1.42**–**1.63)**		
Sex			
Female	1.0	1.0	1.0
Male	**0.62 (0.58**–**0.67)**	0.60 (0.55–1.07)	**0.71 (0.62**–**0.82)**
Age at index date (years)			
20–44			1.0
45–59	**0.81 (0.76**–**0.88)**	**0.84 (0.77**–**0.91)**	**0.76 (0.66**–**0.88)**
≥60	**0.84 (0.75**–**0.93)**	**0.83 (0.74**–**0.94)**	0.84 (0.67–1.05)
Index year			
1994	1.0	1.0	1.0
1995–1998	**1.21 (1.12**–**1.31)**	**1.16 (1.07**–**1.27)**	**1.41 (1.20**–**1.67)**
1999–2002	**1.40 (1.28**–**1.53)**	**1.31 (1.19**–**1.46)**	**1.75 (1.46**–**2.09)**
2003–2006	**1.36 (1.21**–**1.52)**	**1.22 (1.06**–**1.40)**	**1.83 (1.47**–**2.28)**
≥2007	**1.68 (1.41**–**1.99)**	**1.62 (1.32**–**2.00)**	**1.91 (1.41**–**2.59)**
Socioeconomic status			
Quintile 1 (lowest)	1.0	1.0	1.0
Quintile 2	1.03 (0.95–1.13)	0.97 (0.88–1.07)	**1.26 (1.05**–**1.51)**
Quintile 3	1.08 (0.98–1.19)	1.05 (0.94–1.17)	1.20 (0.98–1.46)
Quintile 4	**1.11 (1.01**–**1.22)**	1.05 (0.94–1.18)	**1.30 (1.07**–**1.59)**
Quintile 5	1.06 (0.95–1.17)	0.99 (0.88–1.12)	**1.28 (1.04**–**1.58)**
Physical Comorbidity			
Diabetes	1.11 (0.99–1.25)	1.09 (0.95–1.24)	1.22 (0.94–1.58)
Hypertension	**1.17 (1.08**–**1.27)**	**1.19 (1.08**–**1.31)**	1.06 (0.89–1.27)
Ischemic heart disease	**1.63 (1.45**–**1.83)**	**1.67 (1.47**–**1.91)**	**1.46 (1.11**–**1.93)**
Hyperlipidemia	**1.19 (1.09**–**1.30)**	**1.18 (1.07**–**1.31)**	1.18 (0.96–1.45)
Lung disease	**1.46 (1.33**–**1.59)**	**1.40 (1.27**–**1.55)**	**1.60 (1.34**–**1.90)**
Fibromyalgia	**1.94 (1.62**–**2.31)**	**2.04 (1.63**–**2.55)**	**1.85 (1.38**–**2.48)**
Epilepsy	**1.56 (1.27**–**1.92)**	**1.95 (1.48**–**2.57)**	1.30 (0.96–1.77)
Inflammatory bowel disease	**1.60 (1.10**–**2.32)**	**1.62 (1.04**–**2.52)**	1.48 (0.74–2.98)

a All physical comorbidities included simultaneously in the multivariable models.

b Main model. All main effects for comorbidity statistically significant after using Benjamini–Hochberg correction. Interactions between population and hypertension, epilepsy also statistically significant.

c Stratified models illustrate interaction effects.

Bold text indicates findings that are statistically significant.

We observed interactions between population and hypertension (*χ* ^2^ = 5.82, *P* = 0.02), and epilepsy (*χ* ^2^ = 5.06, *P* = 0.02); therefore we stratified the analysis (Table 3). Hypertension was not associated with increased risk of anxiety disorders in the MS population, but was associated with a slightly increased risk in the matched population. Epilepsy was associated with a nonsignificant 1.3‐fold increased risk in the MS population but a nearly twofold increased risk of anxiety disorders in the matched population. IHD, lung disease, and fibromyalgia were associated with increased risk of anxiety disorders in both populations. In the model combining both populations, hyperlipidemia and IBD were also associated with anxiety disorders. Although we did not detect an interaction between study population and these conditions, the association did not reach statistical significance in the MS population alone.

We did not observe an interaction between sex and population (*χ* ^2^ = 3.46, *P* = 0.06), but found an interaction between sex and fibromyalgia (*χ* ^2^ = 3.88, *P* = 0.05), with the risk of anxiety disorder higher among men (HR 2.55; 1.79–3.64) than women (HR 1.79; 1.46–2.19).

### Physical comorbidity and bipolar disorder

On univariate analysis, the risk of incident bipolar disorder was over twofold higher in the MS population than in the matched population (HR 2.74; 2.36–3.18). The association persisted after adjusting for covariates including physical comorbidities (HR 2.67; 2.29–3.11). The risk of bipolar disorder declined with increasing age (Table 4).

**Table 4:** Adjusted hazard ratios and 95% confidence intervals (HR) for the association between physical comorbidity^a^ and bipolar disorder in the multiple sclerosis (MS) and matched populations

Variable	Both populations^b^ (*N* = 50,715)	Matched population only^c^ (*N* = 40,906)	MS population only^c^ (*N* = 9539)
Population			
Matched	1.0	–	–
MS	**2.67 (2.29**–**3.11)**		
Sex			
Female	1.0	1.0	1.0
Male	1.06 (0.91–1.24)	0.94 (0.77–1.15)	1.29 (1.01–1.66)
Age at index date (years)			
20–44	1.0	1.0	1.0
45–59	**0.68 (0.56**–**0.81)**	**0.74 (0.59**–**0.94)**	**0.61 (0.45**–**0.82)**
≥60	0.79 (0.60–1.02)	0.96 (0.71–1.31)	**0.39 (0.21**–**0.72)**
Index year			
1994	1.0	1.0	1.0
1995–1998	1.02 (0.85–1.24)	0.98 (0.78–1.24)	1.11 (0.80–1.54)
1999–2002	**1.35 (1.08**–**1.68)**	**1.34 (1.02**–**1.77)**	1.40 (0.96–2.03)
2003–2006	**1.48 (1.11**–**1.98)**	1.20 (0.81–1.79)	**1.94 (1.26**–**2.99)**
≥2007	**3.02 (2.08**–**4.38)**	**2.54 (1.48**–**4.34)**	**3.70 (2.17**–**6.30)**
Socioeconomic status			
Quintile 1	1.0	1.0	1.0
Quintile 2	**0.78 (0.63**–**0.96)**	**0.60 (0.46**–**0.78)**	1.21 (0.84–1.72)
Quintile 3	0.99 (0.80–1.23)	0.91 (0.70–1.18)	1.21 (0.82–1.77)
Quintile 4	**0.67 (0.52**–**0.85)**	**0.54 (0.40**–**0.74)**	0.96 (0.64–1.45)
Quintile 5	0.81 (0.63–1.04)	**0.68 (0.50**–**0.93)**	1.14 (0.75–1.72)
Physical Comorbidity			
Diabetes	**1.59 (1.24**–**2.04)**	**1.59 (1.19**–**2.13)**	1.57 (0.97–2.54)
Hypertension	1.08 (0.88–1.33)	1.06 (0.83–1.36)	1.06 (0.74–1.52)
Ischemic heart disease	**1.34 (1.00**–**1.80)**	**1.52 (1.09**–**2.11)**	0.81 (0.40–1.66)
Hyperlipidemia	0.93 (0.74–1.17)	0.94 (0.72–1.23)	0.88 (0.56–1.37)
Lung disease	**1.67 (1.37**–**2.04)**	**1.71 (1.34**–**2.17)**	**1.57 (1.12**–**2.20)**
Fibromyalgia	1.01 (0.61–1.67)	1.61 (0.90–2.88)	0.52 (0.19–1.41)
Epilepsy	**1.83 (1.23**–**2.72)**	**3.11 (1.82**–**5.32)**	1.31 (0.73–2.34)
Inflammatory bowel disease	**2.45 (1.22**–**4.92)**	1.56 (0.50–4.86)	**3.39 (1.39**–**8.24)**

a All physical comorbidities included simultaneously in the multivariable models.

b Main model. All main effects for comorbidity statistically significant after using Benjamini–Hochberg correction. Interactions between population and ischemic heart disease, epilepsy also statistically significant.

c Stratified models illustrate interaction effects.

Bold text indicates findings that are statistically significant.

Because we observed interactions between population and IHD, (*χ* ^2^ = 8.48, *P* = 0.004) and between population epilepsy (*χ* ^2^ = 7.05, *P* = 0.008), we stratified the analysis (Table 4). IHD and epilepsy were not associated with bipolar disorder in the MS population but were associated with an increased risk of bipolar disorder in the matched population. Diabetes was associated with bipolar disorder in both populations to a similar extent although this was marginally nonsignificant in the MS population. Lung disease was associated with bipolar disorder in both populations, whereas, IBD was associated with a threefold increased risk of bipolar disorder only in the MS population.

We neither observed evidence of an interaction between sex and population (*χ* ^2^ = 3.08, *P* = 0.08), nor between sex and any of the physical comorbidities (*P* > 0.05).

## Discussion

We found that the MS population had an increased incidence of psychiatric disorders versus the matched population. However, physical comorbidity was independently associated with an increased risk of incident psychiatric disorders in both populations and contrary to our hypothesis, the increased risk of incident psychiatric disorders in the MS population was not attenuated after accounting for physical comorbidities. Quantifying and disentangling these relationships is important to understand how to address the problem of psychiatric comorbidity in MS.

As expected, the MS population had an increased incidence of depression, anxiety disorders, and bipolar disorder as compared to the matched population (Marrie et al. 2015a), and the incidence of depression and anxiety disorders was lower in men than women (Steel et al. 2014), and lower at older rather than younger ages in both populations (Marrie et al. 2015d). Previously, we had found the disparity in the risk of depression between men and women to be smaller in the MS population than a matched population in other Canadian provinces (Marrie et al. 2015d), findings also seen in a population‐based national Canadian survey (Patten et al. 2003). Collectively, these findings indicate that MS elevates the risk of depression more in men than women.

Chronic lung disease, IHD, and fibromyalgia were associated with an increased incidence of depression and anxiety disorders in both populations. All other comorbidities were associated with depression in the matched population, and all but diabetes were associated with anxiety. Though their associations with depression did not reach statistical significance in the MS population, the magnitudes and directions of association for diabetes and IBD in the MS population were similar to those of the matched population and to those reported previously (Marrie et al. 2009); the same was true for epilepsy and anxiety disorders. In general, persons with chronic medical conditions have an increased risk of depression (Patten 2001). Anxiety disorders are common in the presence of various chronic conditions including arthritis, cancer, lung disease, and neurologic disorders (Wells et al. 1988), while fibromyalgia, epilepsy, and IHD are associated with increased prevalence of both depression and anxiety disorders (Weir et al. 2006; Tellez‐Zenteno et al. 2007; Janssens et al. 2015).

Lung disease was associated with an increased risk of bipolar disorder in both populations. Diabetes and IHD were also associated with bipolar disorder in the matched population and while their associations did not reach statistical significance in the MS population, they were of similar magnitude nonetheless. Others have reported an association between autoimmune disease and bipolar disorder; including a Danish study that found that Guillain–Barre syndrome, Crohn's disease, and autoimmune hepatitis were associated with an increased risk of bipolar disorder (Eaton et al. 2010). In an American study, elevated anti‐Saccharomyces cerevisiae antibodies, a marker of gastrointestinal inflammation, were associated with an increased risk of bipolar disorder (Severance et al. 2014). A Taiwanese study using administrative data found a twofold increased risk of bipolar disorder among individuals with RA in whom asthma, liver cirrhosis, and substance abuse were additional independent risk factors for bipolar disorder (Hsu et al. 2014). While bipolar disorder is associated with an increased prevalence of type 2 diabetes, obesity, and cardiovascular disease in the general population (Rosenblat and McIntyre 2015), prior studies have not evaluated whether these conditions increase the risk of bipolar disorder. To the best of our knowledge, no prior studies have evaluated whether physical comorbidities increase the risk of bipolar disorder in the MS population; thus, our novel findings require confirmation.

Several physical comorbidities were associated with a nonspecific increased risk of two or more of the psychiatric disorders evaluated, implying common mechanisms of effect across disorders. Emerging evidence suggests that inflammation and activation of cell‐mediated immunity contribute to the risk of depression (Lotrich et al. 2011), anxiety disorders (Furtado and Katzman 2015), and bipolar disorder (Rosenblat and McIntyre 2015); thus, the inflammation associated with MS and with diseases such as diabetes (Spranger et al. 2003), chronic lung disease (Fahy 2015), or IBD, may promote psychiatric disorders in susceptible individuals. This susceptibility may reflect a common genetic factor; for example, a recent population‐based Swedish study suggested that a general genetic factor influenced the risk of eight psychiatric disorders, including those we evaluated (Pettersson et al. 2015). While the effects of physical comorbidity on the risk of psychiatric disorders were at times less in the MS population than in the matched population, this could reflect a threshold effect because of an already elevated risk of psychiatric comorbidity in MS.

Notably, we found that sex modified the effect of physical comorbidity on the risk of depression, and of anxiety. Similar to our finding that MS conferred a higher relative risk of depression among men than among women (Table 2), diabetes, hypertension, and lung disease also conferred higher relative risks of depression among men (Table S3). Fibromyalgia conferred a higher relative risk of anxiety disorders among men than among women. Prior work evaluating sex‐specific risks of psychiatric disorders secondary to chronic disease has been limited. In the Canadian National Population Health Survey, hypertension conferred an increased risk of depression only among men (Patten 2001). Some other studies reported more depressive symptoms among men with fibromyalgia than women (Buskila et al. 2000), but not consistently (Häuser et al. 2011). Sex‐specific differences in disease risk may reflect biological or sociocultural factors. For example, female smokers may be at increased risk of chronic obstructive pulmonary disease than male smokers (Sin et al. 2007) due to biological factors such as hormonal effects. However, social support and access to health services may also differ between men and women (Jensen et al. 2014), contributing to differential risk of disease.

Strengths of this study include that it is large, population‐based, used concurrent controls, and used validated approaches to identifying the MS population and comorbidity. However, limitations should be recognized. We conducted this study in one jurisdiction; however, our findings regarding the association of psychiatric comorbidity with MS are similar to those reported in other Canadian jurisdictions. Although we used validated case definitions, administrative data lack clinical details and are not collected for research; so, misclassification may occur. Comorbidity may have been ascertained more readily in the MS population due to frequent health system contacts, but this would not be expected to affect the associations within study populations. We did not assess all potentially relevant comorbidities. The age at MS diagnosis was later than sometimes reported, which may reflect exclusion of individuals under age 20 and inclusion of prevalent cases (e.g., identified when our data began in the 1990s or when they immigrated into the province). However, other studies have reported similar later ages at diagnosis (Fromont et al. 2013). MS is a heterogeneous disease and we did not evaluate the clinical characteristics of MS or its treatment as confounders or effect modifiers of the associations studied. We considered psychiatric disorders as comorbidities, but arguably these conditions may be considered as symptoms or complications in some cases, given their association with structural changes in the brain (Feinstein et al. 2004). Finally, given observations in the general population (Wu et al. 2012; Chien et al. 2013), the association between psychiatric and physical comorbidities in MS is likely bidirectional, but we did not evaluate the risk of physical comorbidity conferred by psychiatric comorbidity herein.

MS was associated with an increased risk of incident depression, anxiety disorders, and bipolar disorder compared to a matched general population. Multiple physical comorbidities were associated with an increased incidence of depression, anxiety disorders, or bipolar disorder within the MS and matched populations. However, the increased risk of psychiatric comorbidity among the MS population was not attenuated after accounting for physical comorbidities, suggesting that these conditions do not account for the differential risk of psychiatric disorders between the MS and matched populations. Regardless, in the MS population, chronic lung disease was associated with a nonspecific increased incidence of depression, anxiety disorders, and bipolar disorder while IHD and fibromyalgia were associated with increased risk of depression and anxiety disorders. Clinicians should be aware that persons with MS are at increased risk of psychiatric comorbidity in general, and that some specific physical comorbidities are associated with additional risk.

## Conflict of Interest

Ruth Ann Marrie receives research funding from: Canadian Institutes of Health Research, Research Manitoba, Multiple Sclerosis Society of Canada, Multiple Sclerosis Scientific Foundation, National Multiple Sclerosis Society, Rx & D Health Research Foundation, and has conducted clinical trials funded by Sanofi‐Aventis. Sharon Warren receives research funding from the CIHR. Christina Wolfson receives research funding from the Multiple Sclerosis Society of Canada, Canadian Institutes of Health Research, Canada Foundation for Innovation, and the National Multiple Sclerosis Society. She has received a speaking honorarium from Novartis Pharmaceuticals. Nathalie Jette is the holder of a Canada Research Chair in Neurological Health Sciences and holds research grants from the Canadian Institutes of Health Research, Alberta Innovates Health Solutions, the Alberta Spine Foundation, the Hotchkiss Brain Institute (HBI), Cumming School of Medicine, University of Calgary, and a shared grant from the HBI/Pfizer Canada. She is a member of the Editorial Board of Neurology. Scott Patten has received honoraria for reviewing investigator‐initiated grant applications submitted to Lundbeck and Pfizer and has received speaking honoraria from Teva and Lundbeck. He is the recipient of a salary support award (Senior Health Scholar) from Alberta Innovates, Health Solutions and receives research funding from the Canadian Institutes for Health Research, Alberta Health Services and the Alberta Collaborative Research Grants Initiative. Helen Tremlett is funded by the Multiple Sclerosis Society of Canada (Don Paty Career Development Award); is a Michael Smith Foundation for Health Research Scholar and the Canada Research Chair for Neuroepidemiology and Multiple Sclerosis. She has received research support from the National Multiple Sclerosis Society, the Canadian Institutes of Health Research, and the UK MS Trust; speaker honoraria and/or travel expenses to attend conferences from the Consortium of MS Centres (2013), the National MS Society (2012, 2014), Bayer Pharmaceuticals (2010), Teva Pharmaceuticals (2011), ECTRIMS (2011, 2012, 2013, 2014), UK MS Trust (2011), the Chesapeake Health Education Program, US Veterans Affairs (2012), Novartis Canada (2012), Biogen Idec (2014), American Academy of Neurology (2013, 2014, 2015). Unless otherwise stated, all speaker honoraria are either donated to an MS charity or to an unrestricted grant for use by her research group. John Fisk receives funding from the Canadian Institutes of Health Research (CIHR), MS Society of Canada and National Multiple Sclerosis Society and has received speaker honoraria from EMD Serono (2013, 2014).

## Supporting information


**Table S1.** Average annual age‐specific incidence of psychiatric comorbidity in the multiple sclerosis and matched populations.
**Table S2.** Average annual sex‐specific incidence of psychiatric comorbidity in the multiple sclerosis and matched populations.
**Table S3.** Association of diabetes, hypertension, and chronic lung disease with depression, stratified by sex (both populations).
**Figure S1.** Annual incidence of depression in the multiple sclerosis (MS) and matched populations, 1999‐2011.
**Figure S2.** Annual incidence of anxiety in the multiple sclerosis (MS) and matched populations, 1999‐2011.
**Figure S3.** Annual incidence of bipolar disorder in the multiple sclerosis (MS) and matched populations, 1999‐2011.
