# Supplementary material for: Physical comorbidities increase the risk of psychiatric comorbidity in multiple sclerosis
Source: Brain Behav. 2016 Jun 29;6(9):e00493. doi: 10.1002/brb3.493 (PMC5036426; doi:10.1002/brb3.493)
Supplement: Supplementary file 1 — Table S1. Average annual age‐specific incidence of psychiatric comorbidity in the multiple sclerosis and matched populations. Table S2. Average annual sex‐specific incidence of psychiatric comorbidity in the multiple sclerosis and matched populations. Table S3. Association of diabetes, hypertension, and chronic lung disease with depression, stratified by sex (both populations). Figure S1. Annual incidence of depression in the multiple sclerosis (MS) and matched populations, 1999‐2011. Figure S2. Annual incidence of anxiety in the multiple sclerosis (MS) and matched populations, 1999‐2011. Figure S3. Annual incidence of bipolar disorder in the multiple sclerosis (MS) and matched populations, 1999‐2011. [file BRB3-6-e00493-s001.doc]

Table e-1 Average annual age-specific incidence of psychiatric comorbidity in the multiple sclerosis and matched populations

| ***Multiple Sclerosis Population*** | | | | |
| --- | --- | --- | --- | --- |
| **Age Group** | **Depression**  **(95% CI)** | **Anxiety**  **(95% CI)** | **Bipolar Disorder**  **(95% CI)** | |
| 20-44 | 3739.5  (3524.4, 3967.7) | 2255.6  (2099.6, 2423.3) | 736.3  (657.2, 825.0) | |
| 45-59 | 2981.6  (2791.8, 3184.3) | 1739.1  (1606.9, 1882.1) | 649.0  (578.6, 728.1) | |
| 60+ | 2384.9  (2149.9, 2645.6) | 1629.1  (1446.2, 1835.1) | 299.2  (233.3, 383.8) | |
| Total | 3160.7  (3035.2,3291.4) | 1918.6  (1827.7, 2013.9) | 613.8  (568.4, 662.9) | |
| ***Matched Population*** | | | | |
| **Age Group** | **Depression**  **(95% CI)** | **Anxiety**  **(95% CI)** | | **Bipolar Disorder**  **(95% CI)** |
| 20-44 | 1690.1  (1629.4, 1753.1) | 1253.7  (1203.1, 1306.4) | | 255.0  (233.9, 278.0) |
| 45-59 | 1407.8  (1355.8, 1461.9) | 1127.5  (1082.5, 1174.3) | | 245.1  (226.0, 265.8) |
| 60+ | 1367.8  (1298.2, 1441.1) | 1134.1  (1072.1, 1199.7) | | 203.1  (179.5, 229.8) |
| Total | 1501.99  (1467.2, 1537.6) | 1175.1  (1145.2, 1205.7) | | 239.4  (226.9, 252.5) |

Table e-2 Average annual sex-specific incidence of psychiatric comorbidity in the multiple sclerosis and matched populations

| ***Multiple Sclerosis Population*** | | | | |
| --- | --- | --- | --- | --- |
|  | **Depression**  **(95% CI)** | **Anxiety**  **(95% CI)** | **Bipolar Disorder**  **(95% CI)** | |
| Women | 3382.4  (3227.1, 3545.1) | 2090.9  (1978.2, 2210.1) | 597.2  (545.0, 654.4) | |
| Men | 2638.7  (2435.1, 2859.3) | 1502.9  (1359.2, 1661.9) | 599.7  (516.9, 695.7) | |
| RR | 1.28  (1.17, 1.41) | 1.39  (1.24, 1.56) | 1.0  (0.84, 1.19) | |
| ***Matched Population*** | | | | |
|  | **Depression**  **(95% CI)** | **Anxiety**  **(95% CI)** | | **Bipolar Disorder**  **(95% CI)** |
| Women | 1708.5  (1664.0, 1754.2) | 1349.8  (1311.7, 1389.1) | | 247.6  (232.8, 263.4) |
| Men | 1028.9  (977.4, 1083.0) | 762.9  (719.5, 808.9) | | 189.6  (169.3, 212.4) |
| RR | 1.66  (1.57, 1.76) | 1.77  (1.66, 1.89) | | 1.31  (1.15, 1.49) |

Table e-3. Association of diabetes, hypertension and chronic lung disease with depression, stratified by sex (both populations)

| **Comorbidity** | **Women** | **Men** |
| --- | --- | --- |
| Diabetes | 1.09 (0.96-1.23) | 1.31 (1.11-1.55) |
| Hypertension | 1.14 (1.04-1.23) | 1.25 (1.09-1.43) |
| Lung disease | 1.46 (1.34-1.58) | 1.69 (1.46-1.96) |

Interactions with sex: diabetes χ^2^ = 5.72, p=0.02, hypertension χ^2^ = 4.94, p=0.03 and chronic lung disease χ^2^ = 4.56, p=0.03

Figure e1. Annual incidence of depression in the multiple sclerosis (MS) and matched populations, 1999-2011

Figure e2. Annual incidence of anxiety in the multiple sclerosis (MS) and matched populations, 1999-2011

Figure e3. Annual incidence of bipolar disorder in the multiple sclerosis (MS) and matched populations, 1999-2011
